# A systematic evaluation of compliance and reporting of patient-reported outcome endpoints in ovarian cancer randomised controlled trials: implications for generalisability and clinical practice

**DOI:** 10.1186/s41687-017-0008-3

**Published:** 2017-10-04

**Authors:** Rebecca Mercieca-Bebber, Michael Friedlander, Melanie Calvert, Martin Stockler, Derek Kyte, Peey-Sei Kok, Madeleine T. King

**Affiliations:** 1 Central Clinical School, Sydney Medical School, University of Sydney, Sydney, NSW Australia; 2 Psycho-oncology Co-operative Research Group, School of Psychology, University of Sydney, Level 6 North, Chris O’Brien Lifehouse C39Z, Sydney, NSW Australia; 3 NHMRC Clinical Trials Centre, University of Sydney, Camperdown, NSW Australia; 4 Australian New Zealand Gynecological Oncology Group (ANZGOG), Camperdown, NSW Australia; 5 Centre for Patient-Reported Outcomes Research, University of Birmingham, Edgbaston, Birmingham, UK

**Keywords:** Quality of life, Patient-reported outcomes, Missing data, Reporting, Ovarian neoplasms

## Abstract

**Background:**

This study aimed to evaluate the patient-reported outcome (PRO) content of ovarian cancer randomised-controlled trial (RCT) publications, describe PRO compliance, and explore potential relationships among these and completeness of PRO protocol content.

**Methods:**

Publications of Phase III ovarian cancer RCTs with PRO endpoints were identified by Medline and Cochrane systematic search: January 2000 to February 2016. Two reviewers determined the number of Consolidated Standards of Reporting Trials (CONSORT)-PRO Extension items addressed in publications. Compliance rates (defined as the proportion of participants included in the principal PRO analysis, of those from whom PRO assessments were expected) were extracted. The relationship between CONSORT-PRO score and compliance rates was explored using scatter plots. Additionally CONSORT-PRO score and PRO compliance rates respectively were compared with corresponding PRO protocol scores obtained from a previous study.

**Results:**

Thirty-six eligible RCTs (*n* = 33 with secondary PRO endpoint) were identified and analysed. The average number of CONSORT-PRO items addressed in publications was 6.7 (48%; Range 0–13.5/14). Three RCTs did not report PRO results; in 1 case due to poor compliance. Some compliance information was reported in 26 RCTs, but was considered complete for only 10 (28%) RCTs. Compliance rates were poor overall, ranging from 59 to 83%; therefore missing PRO data from 17 to 41% of participants in these trials could have been avoided.

Of the 26 (73%) RCTs for which PRO protocol completeness scores were available, 6 RCTs reported complete compliance information and the 3 of these RCTs with highest PRO compliance had highest protocol checklist scores.

**Conclusions:**

Few RCTs reported PRO compliance information in a manner enabling assessment of the generalisability of PRO results. This information is particularly important in RCTs of advanced ovarian cancer because it is important to be able to determine if missing data was due to worsening illness compared to methodological issues. Poor compliance appeared related to poor PRO protocol content, and in one case prevented PRO results from being reported, highlighting the need to address compliance strategies in the protocol. Adhering to protocol and CONSORT-PRO reporting guidance should improve PRO implementation and reporting respectively in ovarian cancer RCTs and allow results to meaningfully inform clinical practice.

**Electronic supplementary material:**

The online version of this article (doi:10.1186/s41687-017-0008-3) contains supplementary material, which is available to authorized users.

## Background

Patient-reported outcomes (PROs), including health-related quality of life (HRQOL), assessed in cancer randomised controlled trials (RCTs) provide valuable information on the impact of treatment from the patient’s perspective [1, 2]. PRO data are increasingly being used to inform clinical practice guidelines, policy decisions and therapeutic labelling, with growing support by professional oncology societies, including European Medical Association (EMA) [3], American Society of Clinical Oncology (ASCO) [4], and European Society for Medical Oncology (ESMO) [5] to incorporate PROs in the comprehensive assessment of clinical benefit.

PROs have long been included as secondary endpoints in ovarian cancer RCTs [6], as treatment is often associated with significant adverse effects, particularly in patients with platinum resistant ovarian cancer with poor performance status and high symptom burden [7]. In patients with symptomatic recurrent ovarian cancer, chemotherapy can also palliate symptoms [7]. ESMO recommends that the primary endpoint of treatment for these patients should be symptom control and HRQOL [8]. The importance of evaluating PROs in ovarian cancer RCTs has also been highlighted by the 3rd [9] and 4th [10, 11] Ovarian Cancer Consensus meetings and the Food and Drug Administration (FDA) 2006 Ovarian Cancer Endpoints Workshop [12]. Yet shortcomings in PRO trial design, methodology and reporting may limit the interpretation of PRO data and its potential to inform patient-centred care [13].

A comprehensive RCT protocol with well-considered PRO hypotheses is essential to communicate the rationale and methods for high-quality PRO data collection. It is therefore concerning that recent evidence indicates trial staff perceive PRO data collection guidance to be inadequate [14]. Insufficient PRO data collection guidance may lead to inconsistent procedures [15], missing PRO data [16] and potentially misinterpretation of PRO findings if the RCT publication does not clearly discuss the associated potential for bias.

Adding to the complexity of PRO interpretation in oncology is missing data, which may be unavoidable if related to disease progression or death; in such cases missing data is considered to be “informative” of poor health status [17]. However participants who are alive and still enrolled in the trial may also have missing PRO data, potentially due to trial staff ‘gate-keeping’ (deciding that participants are too unwell or that participants should not complete a questionnaire), administrative errors or participant refusal, which may be avoidable in many cases by the provision of high-quality trial guidance or staff training in the trial protocol [16, 18]. In any case, reasons for missing data should inform appropriate selection of analysis methods and accurate data interpretation [16, 17]. Likewise this information should be reported to assist readers’ interpretation of findings by differentiating between the participants who complete PRO assessments as a proportion of the full sample (intention-to-treat population) as opposed to “PRO assessment compliance”, or the proportion who complete scheduled PRO assessments of those from whom PRO assessments are expected (participants still enrolled on the trial) [19]. The latter is considered an indicator of the efficacy of design and methodological strategies in preventing avoidable missing data for the purpose of this study because by definition it acknowledges that PRO assessments cannot be expected from deceased or withdrawn participants.

Another challenge for the uptake of PRO evidence from RCTs is poor PRO reporting according to CONSORT (CONsolidated Standards for Reporting) reporting standards [20–22]. Poor reporting arguably limits the extent to which PRO data can inform clinical practice. To our knowledge, no studies to date have explored associations among PRO protocol content, compliance and reporting.

For any RCT, both the protocol and resultant publication should clearly articulate PRO hypotheses, endpoints, methodology and analyses. This is reflected in overlapping content of the CONSORT-PRO [20] and PRO-specific guidance for protocols currently in development: SPIRIT-PRO extension (Standardised Protocol Items for Randomised Trials) [23, 24]. Given the parallels in desirable content of RCT publications and protocols, we sought to study links between protocol and publication content.

In our earlier study [25], we established that the PRO content of 26 ovarian cancer RCT protocols was often suboptimal when assessed against a checklist of recommended items. On average, protocols addressed less than 1/3 of recommended PRO items, with most trials offering only basic information, such as the PRO assessment schedule and questionnaires used [25]. PRO-specific quality assurance procedures were generally lacking, as were procedures for explaining the purpose of PRO assessments to participants, following up missed assessments and handling missing data in the analysis [25]; all of which have been identified as important for minimising the problem of missing PRO data [16] and for overall protocol completeness [24] suggesting a potential relationship between PRO content of trial protocols and PRO compliance.

The aims of this study were to describe the quality of reporting of PROs in ovarian cancer RCTs based on the CONSORT-PRO Extension; describe PRO compliance rates and the reporting of PRO compliance. We also aimed to explore the relationship between CONSORT-PRO reporting score and other key variables which we thought may influence reporting, including whether there was a significant difference in the primary trial endpoint or the PRO endpoint, compliance rates and year of publication. We also explored whether the PRO content of the ovarian cancer RCT protocols reviewed previously [25] had an impact on: 1) the overall standard of PRO reporting according to the CONSORT-PRO, and 2) PRO compliance. We hypothesised that RCTs with more complete PRO protocol content would have more complete reporting and higher PRO compliance rates.

## Methods

### Identification of RCTs

**Fig. 1:**
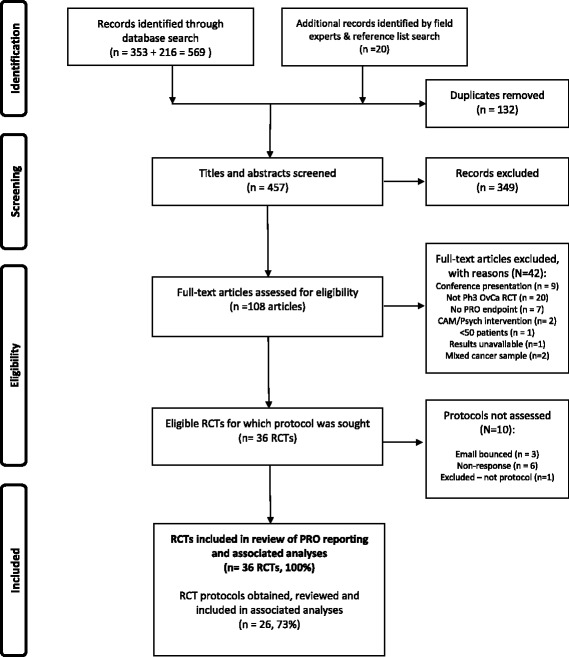
Flow diagram of RCT selection

Our search and selection strategy were published previously [25] and summarised in Fig. 1. Briefly, 36 phase III biomedical ovarian cancer RCTs published between January 2000 and February 2016 were identified by a systematic search of Medline and Cochrane Clinical Trials databases, searching reference lists of eligible RCTs and by consulting the Gynaecologic Cancer Inter-Group (GCIG) Symptom Benefit Working Group (SBWG).

### RCT protocol scoring

We sought the RCT protocol for all 36 RCTs for evaluation in this study by searching online or by contacting the corresponding author of the trial publication. Protocol authors did not have access to the PRO protocol checklist, which minimised the risk of them augmenting their protocols prior to our evaluation.

Two authors (RMB, PK) independently reviewed PRO content of each protocol obtained (*n* = 26, Fig. 1) against a checklist of 58 recommended items (Additional file 1: Appendix 1a) to minimise bias [25]. A total PRO protocol checklist total score (%) was calculated for each protocol, considering only the checklist items directly applicable to each RCT. Scoring discrepancies were resolved upon discussion with a third author (MK, MF or MC).

### RCT publications: CONSORT-PRO scoring and compliance data extraction

Two authors (RMB, PK) independently assessed PRO content of all eligible RCT publications (*n* = 36) against the CONSORT-PRO checklist adapted for review purposes (scoring described in Additional file 1: Appendix 1b), awarding points to each checklist item addressed and combining into an overall total score (maximum 14). If the RCT was reported across multiple publications, we considered all publications together and gave a single score for the RCT.

We defined PRO compliance rate as the proportion of participants included in the principal PRO analysis, of those from whom PRO assessments were expected per protocol (i.e. still on study, still alive) [19]. This level of reporting was considered necessary to minimise risk of bias caused by missing PRO data [16] and is also required by CONSORT-PRO [20]. Two authors (RMB, PK) extracted PRO compliance information:

1. The actual *PRO compliance rate* at the principal time-point of PRO analysis was extracted from publications, or calculated where this was possible based on the information reported in the publication.
2. *Reporting of compliance* was then classified at one of three possible levels: ‘*adequate’* if the RCT reported compliance information according to our definition [19] or provided sufficient information for us to calculate this; ‘*incomplete’* if the number of participants included in PRO analyses or the number of questionnaires completed at each time-point was reported but not the number expected (therefore it was unclear from these publications how many PRO assessments were missing from analyses due to death or worsening illness as opposed to ‘avoidable’ reasons). RCTs that failed to report any of this information were classified as having reported ‘*no compliance information*’. Our grading of compliance reporting did not consider the actual rate of compliance, therefore it was possible for a trial with a poor PRO compliance rate to have ‘adequate’ compliance reporting. Discrepancies were resolved upon discussion (with MK or MC).

### Analyses

Relationships among CONSORT-PRO score, the PRO protocol checklist total score (in the subgroup of 26 RCTs with a protocol), and year of main RCT publication were examined using scatterplots. Dot plots compared total CONSORT-PRO scores for RCTs that reported (versus did not report) a significant difference in either the primary or PRO endpoints.

The potential relationship between PRO compliance rates at the principal time-point of PRO analysis and PRO protocol checklist total scores was assessed with a scatter plot.

The potential relationship between reporting any compliance information (yes vs no) and PRO protocol checklist total score was assessed with dot plots. RCTs with ‘adequate’ and ‘incomplete’ reporting of compliance were pooled (these RCTs made an attempt at reporting compliance) and compared against RCTs reporting ‘no compliance information’.

If any of these visual displays suggested an apparent relationship, an exploratory independent t-test was conducted. All plots and analyses were conducted using SPSS Version 22 (Armonk, NY: IBM Corp).

## Results

### RCT characteristics

Of the 36 eligible RCTs identified (Additional file 2: Appendix 2), 33 had secondary PRO endpoints, 1 had a co-primary PRO endpoint, 1 had a tertiary PRO endpoint and for 1 the PRO endpoint status was unclear (Table 1). Ten RCTs (28%) reported PRO results in a separate publication. Just over half (*n* = 19, 53%) reported a statistically significant difference between treatment groups in at least one PRO scale, 14 (39%) reported no PRO differences, and it was unclear whether there were any PRO differences in the remaining 3 RCTs (12%) as they did not report PRO results.

**Table 1:** Characteristics of the 36 ovarian cancer RCTs

Characteristic		No. of RCTs (%)
Publication in which PRO results published	Main RCT publication	18 (50)
	Dedicated publication	10 (28)
	PROs not published	3 (8)
Year of main RCT publication	2000–2009	15 (42)
	2010–2016	21 (58)
Year of dedicated PRO publication	2000–2009	4 (11)
	2010–2016	6 (17)
	No dedicated QOL publication	26 (72)
PRO endpoint status	Co-primary	1 (3)
	Secondary	33 (96)
	Tertiary	1 (3)
	Unclear	1 (3)
PRO measures used	EORTC QLQ-C30 ^a^	24 (67)
	EORTC QLQ-OV28 ^b^	12 (33)
	FACT-O ^c^	11 (13)
	Other FACIT ^d^ measures	4 (11)
	EQ-5D ^e^	4 (12)
	Other	3 (8)
Sig. difference in primary RCT endpoint	Yes ^f^	15 (42)
	No	21 (81)
Sig. difference in any PRO scale reported	Yes ^f^	19 (53)
	No	14 (39)
	Unclear, as no PRO results reported	3 (8)
Intervention	Chemotherapy	28 (78)
	Targeted therapy	7 (19)
	Surgery	2 (6)
Primary endpoint	Progression-free survival (PFS)	21 (58)
	Overall survival	5 (14)
	Survival (other)	3 (8)
	All-cause mortality	2 (6)
	Time to progression	2 (6)
	PFS and QOL (co-primary)	1 (3)
Sponsors	Clinical Trials Group	25 (69)
	Commercial/pharmaceutical	7 (19)
	Co-sponsored: Trials group and commercial	4 (11)

^a^ European Organisation for Research and Treatment of Cancer (EORTC) Quality of Life Questionnaire-Core 30 (QLQ-C30)

^b^ ovarian cancer module (QLQ-OV28), which is used with the QLQ-C30

^c^ Functional Assessment of Cancer Therapy–Ovarian Cancer Module (FACT-O)

^d^ Functional Assessment of Cancer Therapy (FACIT)

^e^ the EuroQOL-5 dimensions (EQ-5D)

^f^ Includes 1 RCT with co-primary PRO endpoint

### Completeness of PRO reporting

**Fig. 2:**
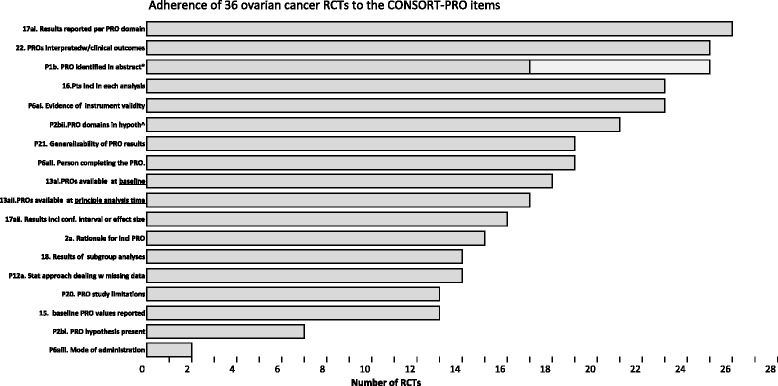
Adherence of 36 ovarian cancer RCTs to CONSORT-PRO items. Legend: *Dark grey shading indicates the abstract identified the PRO as a primary or secondary endpoint, light grey shading indicates the PRO was mentioned in the abstract but endpoint status was unclear. ^Awarded if PRO domains were stated in PRO aims, objectives or hypotheses. CONSORT-PRO items P2b, P6, 13a, 17a and P20/21 contain multiple recommendations and were divided into sub-items for scoring (See Additional file 1: Appendix 1b)

Total CONSORT-PRO scores (*n* = 36) ranged from 0 to 13.5/14, with a mean of 6.7 (48%). Most (*n* = 33, 92%) reported some PRO results. Of the 3 (12%) RCTs that did not report any PRO results, 2 stated that these would be reported subsequently (CONSORT-PRO total scores of 0/14 and 1/14 respectively). The other did not analyse the PRO data due to poor compliance, and did not address any other recommended CONSORT-PRO criteria, scoring 0/14. Another low-scoring publication (scoring 1/14) simply reported that there were no differences in global QOL at any time point, but did not report the time points assessed, analysis methods, or results for other questionnaire domains. The majority of RCTs addressed some CONSORT-PRO items: 27 (75%) RCTs reported results of pre-specified PRO endpoints or all domains of the PRO questionnaire used, 25 (69%) interpreted PROs in the context of clinical endpoints, 19 (53%) provided the number of participants included in each PRO analysis, and 23 (64%) cited evidence of the validity of the PRO questionnaire used. However, other items were reported poorly; most concerning was the limited number of RCTs reporting baseline PROs (*n* = 13, 36%), or reporting approaches for dealing with missing PRO data (*n* = 14, 39%) (Fig. 2).

**Fig. 3:**
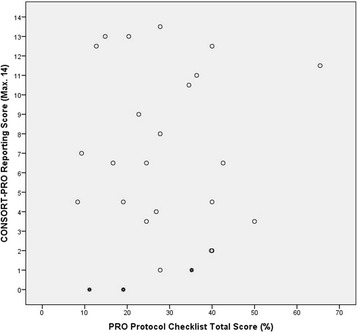
Scatterplot of total CONSORT-PRO score and total PRO Protocol Checklist scores of 26 ovarian cancer RCTs. Legend: PRO results were not published for the 3 RCTs marked with *. The protocols of 10 additional RCTs included in this study were not available for analysis and are excluded from this plot

There was no apparent relationship between the year of publication and CONSORT-PRO score (Additional file 3: Appendix 3a), or CONSORT-PRO score and the PRO protocol checklist total score (Fig. 3); however the 2 RCTs with CONSORT-PRO scores of 0/14 also had PRO protocol scores <20%. There was no apparent difference in CONSORT-PRO scores between RCTs with a significant difference in the primary RCT endpoint, compared to RCTs with no primary significant differences, or in RCTs reporting a significant difference in the PRO endpoint (*n* = 19) compared to RCTs reporting no significant PRO difference (Additional file 3: Appendix 3b).

### Reporting of PRO compliance

Information regarding PRO compliance reported ‘adequately’ by 10 (28%) RCTs, and ‘incompletely’ by 16 (44%) RCTs. The remaining10 (25%) RCTs did not report PRO compliance. Eight of 10 RCTs with adequately reported compliance information addressed >11/14 (79% or higher) CONSORT-PRO items, whilst the remaining 2 scored 3.5 and 4/14. Six (17%) RCTs reported a definition of compliance, all of which were consistent with our definition and all bar one reported compliance ‘adequately’.

Nineteen (53%) RCTs reported the number of baseline questionnaires submitted, and all but 1 of these reported the number submitted for the principal analysis time-point. Six (17%) RCTs reported use of a form to collect reasons for missing PRO data, however, of these, only 3 reported ‘adequate’ compliance information while 3 reported ‘incomplete’ information.

Dot plots showed a wide range in the distribution of PRO protocol checklist scores for RCTs that reported any compliance information compared to RCTs reporting none (Additional file 3: Appendix 3c), however CONSORT-PRO scores were higher for RCTs that reported any compliance information (*t* = 7.56, *p* < .001; Additional file 3: Appendix 3d).

### PRO compliance rates

**Fig. 4:**
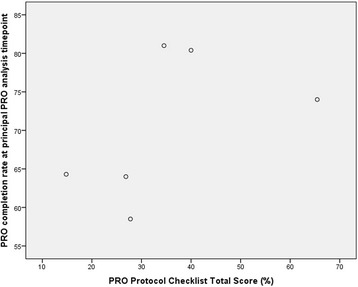
Scatterplot of PRO compliance percentage at the primary PRO analysis timepoint and PRO protocol checklist total score. Legend: PRO compliance rate was clearly reported for 6 of the 26 RCTs for which trial protocols were obtained. The mean PRO protocol score for these 6 RCTs was 34.9%, range: 14.8–65.5%. Of the remaining 20 RCTs with available PRO protocol scores, compliance data was not reported adequately in the publication. The mean PRO protocol score for these 20 RCTs was 26.4%, range 8.3–50%. PRO protocol checklist scores for all 26 RCT protocols are described in detail in [25]

**Fig. 5:**
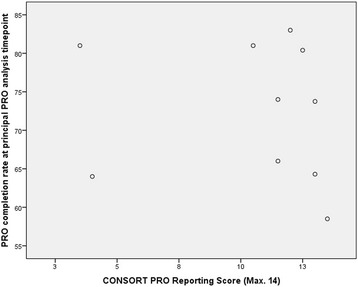
Scatterplot of PRO compliance percentage at the primary PRO analysis timepoint and CONSORT-PRO reporting score. Legend: PRO compliance rate was clearly reported for 10 of the 36 RCTs included in this study. The mean PRO CONSORT-PRO score for these 10 RCTs was 10.5/14, range: 4–13/14. Compliance data was not reported adequately in the remaining 26 RCT publications, therefore these have been excluded here. The mean CONSORT-PRO score for these 26 RCTs was 5.3/14, range 0–13.5/14

Of the 10 RCTs that *adequately* reported PRO compliance, compliance rates ranged from 59 to 83%. We were able to examine the trial protocols of 6 of the 10 RCTs which reported complete compliance information, and those with higher PRO compliance rates at the primary PRO analysis timepoint had higher PRO protocol checklist scores (Fig. 4). However, there was no apparent relationship between compliance rates and CONSORT-PRO scores (Fig. 5) among the 10 RCTs with ‘*adequate’* compliance information.

For 16 RCTs with *incomplete compliance reporting*, it was not possible to determine their exact compliance rates, because it was unclear how many observations were included in the analysis or how many of the missing assessments were due to participant death or withdrawal (unavoidable/informative) as opposed to avoidable reasons. However all these RCTs reported increasing rates of missing PRO data overtime, as is typical in cancer RCTs [26].

One RCT analysed PRO data only from 1 country (representing less than a quarter of overall accrual), due to poor compliance in other countries, though comparisons of the analysed compared to accrued sample were clearly presented and the paper reported compliance ‘adequately’.

## Discussion

This study reviewed the relationship between the completeness of PRO content of ovarian cancer RCT protocols and corresponding publications and PRO compliance rates. There was a large range in CONSORT-PRO scores, much the same as we observed a large variation in corresponding PRO protocol checklist scores previously [25]. On average, RCTs reported less than half the recommended CONSORT-PRO items. The reporting of compliance was particularly poor, with only 10 RCTs (28%) reporting this information adequately. The 3 with the lowest PRO compliance rates had lowest PRO protocol checklist scores, providing some support for one of our motivating hypotheses - that comprehensive consideration of PROs in the protocol will have beneficial effects on subsequent PRO data collection. This highlights the need to address PRO compliance strategies and provide clear PRO data collection guidance in the trial protocol and represents a potential strategy to reduce the risk of missing PRO data in future trials. Compliance rates were poor overall. The highest reported compliance rate was 83%; therefore the 17% of participants with missing data at the principal analysis time-point could have been avoided, possibly through implementation of PRO quality assurance processes [16]. The worst compliance rate reported was 59%, therefore a concerning 41% of participants in that study had avoidably missing data.

Because survival time is short in advanced ovarian cancer [27, 28], trial participants may progress, stop protocol treatment, withdraw from or die during trial follow-up. Additionally, severe chemotherapy toxicity may result in participants feeling too unwell to complete PROs. All of these issues are likely to lead to missing data from participants who have poorer PROs and HRQOL outcomes; “informative” missing PRO data. Indeed, women enrolled in the Australian Ovarian Cancer Study demonstrated sharp declines in QOL preceding drop out and those who dropped out early had poorer QOL at baseline, suggesting an informative missing PRO data pattern [29]. We found that 16 (44%) RCTs reported incomplete compliance information, meaning that despite the authors’ attempts to report information about missing data, it was unclear whether missing data was informative of poor health status or avoidable. In all PRO studies, particularly when compliance is poor, authors need to describe the reasons for missing PRO data and the analysis sample’s representativeness to the target population in line with CONSORT-PRO, to enable the reader to understand the external validity and generalizability of the results [16, 20]. Failing to report the number of participants from whom PROs were expected, and the reasons for missing PRO data, makes it difficult for readers to determine whether missing data are related to poor health status or not, and therefore to assess the generalizability and credibility of the trial findings and conclusions [29].

Statisticians also need to know the reasons for missing PRO data so they may handle it appropriately in analyses [26]. Thus it is important that reasons for missing PRO data are collected in real-time by site staff, so this requirement should be stated in the trial protocol. Yet only 6 (17%) RCTs in this cohort collected this information. Only 2 of these 6 RCTs went on to report compliance information adequately (PRO compliance rates of 74 and 83% respectively at their primary analysis time-points). The remaining 4 RCTs reported compliance incompletely; completion rates among these 4 RCTs ranged from 40 to 51%, however the extent of informatively (unavoidably) missing data was unclear.

We found that 2 RCTs failed to report PRO data due to poor compliance. This represents a waste of research effort as unpublished PRO data cannot possibly be used to improve knowledge or inform clinical practice [15, 30]. Failing to publish PRO data due to poor compliance devalues the contributions and data of compliant participants and centres. Some believe that if PRO compliance falls below a certain threshold, then the data are too unrepresentative to be of use. Whilst this may be true if statistical power is significantly reduced or if remaining participants are grossly unrepresentative of the recruited sample, we argue that transparent reporting of rates and reasons for missing data can inform robust interpretation, if the authors discuss generalisability concerns informed by clinical data collected on these participants [16, 31]. There is no substitute for high PRO completion rates, and trial staff should routinely be reminded of this as a quality assurance measure. However, poor compliance should not always be viewed as a barrier to publication, particularly where missing data was unavoidable and/or informative of poor participant health; the PROs of the compliant participants may still be of interest and value. In the context of ovarian cancer RCTs, the PRO-compliant subgroup is likely to over-represent participants who have stable disease or are responding well to treatment–this is certainly a subgroup of interest, because the QOL of the surviving participants who do well on treatment matters. The results of these participants should be reported as an important sub-group of the recruited sample, *not* as a representative sub-group of the recruited sample, as the latter would present an overly optimistic picture of the effects of the trial treatments (bias). Not reporting any PRO data at all due to poor compliance represents a failure to extract the important evidence in the available data (research waste).

We did not see a relationship overall between protocol content and CONSORT-PRO reporting scores in this sample. Encouragingly this finding suggests that PRO endpoints may reported in line with CONSORT-PRO guidance, even if the trial protocol did not comprehensively address PROs. However, further research is needed as we found that the 2 RCTs that did not report any details on their PRO studies (i.e. CONSORT-PRO scores of 0) addressed less than 20% of recommended PRO-specific protocol items. Non-reporting of PRO results is undoubtedly emerging as a problem in this field. Schandelmaier and colleagues found only 20% of 173 oncology RCTs with PRO endpoints listed in the protocol subsequently went on to publish the PRO data [32]- again demonstrating research waste [33].

Some CONSORT-PRO items were reported by most RCTs, for example 75% reported results by PRO domain and 69% of publications discussed PRO results in the context clinical endpoints, yet the limited number of RCTs that reported baseline PROs (36%) and approaches for missing data (42%) is concerning. Failing to report this information, and incomplete reporting generally, can lead to biased interpretation or make it difficult to assess generalisability of results, which in turn may limit the potential for PROs to impact policy decisions and patient care. It must be noted that all of the RCTs included in our review were designed without the benefit of the yet-to-be-finalised SPIRIT-PRO checklist, and many (69%) were published before the release of CONSORT-PRO guidance in 2013. The deficiencies summarised in this paper underscore the value of those checklists. We hope that by identifying these issues, future investigators will take necessary steps towards improving the quality and dissemination of PRO data collected in RCTs.

Historically, PROs have had a limited role in oncology labelling claims. The earliest FDA drug approval for oncology was in 1995 [34], and by 2006 only a small proportion of oncology drug approvals had included PRO evidence [35]. A more recent critique of ovarian cancer trials reveals that PROs are often omitted or not analysed despite their obvious relevance in this context [13].

Oncology societies and policy makers are now formally recognising the value of PROs. QOL is incorporated into the scoring of the ESMO Magnitude of Clinical Benefit Scale (MCBS) for the management of solid cancers without curative intent, whereby the maximum MCBS score can only be achieved if the therapy “demonstrates improved QOL or delayed deterioration in QOL using a validated scale” as well as superior survival (Cherny, et al., 2015, p1550, [5]). Similarly, ASCO incorporates QOL into the recently-developed Net Health Benefit Framework for advanced disease, which aims to facilitate physician and patient access to information for shared treatment decision-making according to individual patient preferences and circumstances [4]. These initiatives represent real potential for PROs to impact care. PRO researchers must seize this opportunity not only to include PROs when they are relevant, but to improve PRO research practice and, by consequence, the quality and impact of PRO evidence. Therefore PRO studies must be designed, conducted and reported to the highest standards to be of most benefit to patient care. Our findings suggest that: 1) adherence to the forthcoming SPIRIT-PRO Extension and CONSORT-PRO Extension for the development of protocols and publications respectively, and 2) prospectively collecting reasons for missing data and reporting these reasons in the publication, can assist researchers to ensure that high-quality PRO evidence is available and utilised in clinical practice.

### Strengths

This study of all ovarian cancer RCTs published over a 16 year period describes key PRO methodological shortfalls and their potential impact in a clinical sub-group for which PROs are outcomes of high importance. Two authors independently scored protocols and publications, and extracted data. Checklists used to assess protocols and publications were based on current evidence for international best practice in PRO research. We were able to obtain the majority of RCT protocols, even though many of these were not publically available due to their age. RCT authors did not have access to the PRO protocol checklist used, therefore it is unlikely that protocols were augmented prior to our analysis. Specific methodological issues addressed in this review, such as handling and reporting of missing PRO data, are particularly meaningful to ovarian cancer trials and trials of other disease groups.

### Limitations

Although we did not observe a clear relationship between overall reporting completeness and PRO protocol checklist score in ovarian cancer RCTs, this does not rule out such a relationship in oncology RCTs generally. We could not assess whether trial staff adhered to protocol instructions, which may have impacted PRO compliance rates. Our analysis of the relationship between PRO compliance rates with protocol and reporting scores should be interpreted with caution, as this analysis was limited by the small number of trials adequately reporting PRO compliance; however this limitation independently represents an important finding (that compliance is poorly reported) relevant to our aims.

### Next steps

Further work on the impact of PRO protocol content on the quality of data and reporting is needed in other clinical sub-groups for which PROs are of particular importance, and in oncology generally. A similar analysis in a mixed oncology cohort is ongoing as part of the UK Macmillan Cancer Support EPiC study [36]. Relationships between protocols and reporting may emerge in this heterogeneous oncology RCT sample, with a broader variety of PRO and other RCT endpoints, and investigators.

## Conclusions

This study provides preliminary evidence that a trial protocol with more complete details on the PRO endpoint may reduce the risk of avoidable missing PRO data. Poor compliance led to non-reporting of PROs for 2 RCTs, meaning that efforts invested into PRO data collection for these RCTs was wasted as the PRO data cannot possibly impact patient care. It also provides evidence that the reporting of PROs requires improvement, particularly reporting of the rates, reasons and impact of missing PRO data. Given that rates of avoidable and informative missing PRO data were quite high in this sample, clear reporting is crucial and should include a transparent discussion of generalisability concerns in light of avoidable and informative missing data. Investigators should refer to the forthcoming SPIRIT-PRO Extension [23] to develop PRO aspects of trial protocols with clear strategies to minimise the missing data, as well as the CONSORT-PRO guidance for reporting [20]. Such efforts will ensure high-quality PRO findings are accurately interpreted and can meaningfully impact patient care.

## Additional files

Additional file 1: Appendix 1. PRO protocol checklist CONSORT-PRO scoring sheets. (DOCX 27 kb)

Additional file 2: Appendix 2. List of Included RCTs. (DOCX 21 kb)

Additional file 3: Appendix 3. Supplementary plots. (DOCX 135 kb)

## References

[1] Au HJ, Ringash J, Brundage M, Palmer M, Richardson H, Meyer RM (2010). Added value of health-related quality of life measurement in cancer clinical trials: The experience of the NCIC CTG. Expert Review of Pharmacoeconomics & Outcomes Research.

[2] Food and Drug Administration. Guidance for Industry: Patient-Reported Outcome Measures: Use in Medical Product Development to Support Labelling Claims2009. Available from: http://www.fda.gov/downloads/Drugs/Guidances/UCM193282.pdf. Accessed 10 Oct 2016.

[3] European Medicines Agency. (2016). Appendix 2 to the guideline on the evaluation of anticancer medicinal products in man: The use of patient-reported outcome (PRO) measures in oncology studies. London, UK. http://www.ema.europa.eu/docs/en_GB/document_library/Other/2016/04/WC500205159.pdf. Accessed 10 Oct 2016.

[4] Schnipper LE, Davidson NE, Wollins DS, Blayney DW, Dicker AP, Ganz PA, et al. (2016). Updating the American Society of Clinical Oncology Value Framework: Revisions and Reflections in Response to Comments Received. *Journal of Clinical Oncology, 34*(24), 2925–2934. doi:10.1200/JCO.2016.68.2518 10.1200/JCO.2016.68.2518 27247218

[5] Cherny NI, Sullivan R, Dafni U, Kerst JM, Sobrero A, Zielinski C (2015). A standardised, generic, validated approach to stratify the magnitude of clinical benefit that can be anticipated from anti-cancer therapies: The European Society for Medical Oncology magnitude of clinical benefit scale (ESMO-MCBS). Annals of Oncology.

[6] Hess LM, Stehman FB (2012). State of the science in ovarian cancer quality of life research: A systematic review. International Journal of Gynecological Cancer.

[7] Friedlander ML, King MT (2013). Patient-reported outcomes in ovarian cancer clinical trials. Annals of Oncology.

[8] Ledermann JA, Raja FA, Fotopoulou C, Gonzalez-Martin A, Colombo N, Sessa C (2013). Newly diagnosed and relapsed epithelial ovarian carcinoma: ESMO clinical practice guidelines for diagnosis, treatment and follow-up. Annals of Oncology.

[9] du Bois, A., Quinn, M., Thigpen, T., Vermorken, J., Avall-Lundqvist, E., Bookman, M., et al. (2004, 2005, 16). Consensus statements on the management of ovarian cancer: Final document of the 3rd international gynecologic cancer intergroup ovarian cancer consensus conference (GCIG OCCC 2004). *Annals of Oncology*, (Suppl 8), viii7–viii12. 10.1093/annonc/mdi961 16239238

[10] Friedlander M, Trimble E, Tinker A, Alberts D, Avall-Lundqvist E, Brady M (2011). Clinical trials in recurrent ovarian cancer. International Journal of Gynecological Cancer.

[11] Stuart GC, Kitchener H, Bacon M, du Bois A, Friedlander M, Ledermann J (2011). 2010 gynecologic cancer inter group (GCIG) consensus statement on clinical trials in ovarian cancer: Report from the fourth ovarian cancer consensus conference. International Journal of Gynecological Cancer.

[12] Food and Drug Administration. (2006). Ovarian Cancer Endpoints Workshop April 26 2006 meeting summary. Available from: https://www.fda.gov/downloads/AboutFDA/CentersOffices/CDER/ucm120657.pdf. Accessed 9 Aug 2016.

[13] Friedlander M, Mercieca-Bebber R, King M (2016). Patient reported outcomes in ovarian cancer clinical trials-lost opportunities and lessons learned. Annals of Oncology.

[14] Kyte D, Ives J, Draper H, Keeley T, Calvert M (2013). Inconsistencies in quality of life data collection in clinical trials: A potential source of bias? Interviews with research nurses and Trialists. Plos One.

[15] Ioannidis JPA, Greenland S, Hlatky MA, Khoury MJ, Macleod MR, Moher D (2014). Increasing value and reducing waste in research design, conduct, and analysis. The Lancet.

[16] Mercieca-Bebber, R., Palmer, M. J, Brundage, M., Calvert, M., Stockler, M. R., King, M. T., (2016). Design, implementation and reporting strategies to reduce the instance and impact of missing patient-reported outcome (PRO) data: a systematic review. *BMJ Open, 6*(6). 10.1136/bmjopen-2015-010938 PMC4916640 27311907

[17] Fairclough DL, Peterson HF, Chang V (1998). Why are missing quality of life data a problem in clinical trials of cancer therapy?. Statistics in Medicine.

[18] Bernhard, J., Cella DF, Coates, A. S., Fallowfield, L., Ganz, P. A, Moinpour, C. M, et al. (1998). Missing quality of life data in cancer clinical trials: Serious problems and challenges. *Statistics in Medicine, 17*(5–7), 517–532. 10.1002/(sici)1097-0258(19980315/15)17:5/7<517::aid-sim799>3.0.co;2-s 9549801

[19] Osoba D, Bezjak A, Brundage M, Zee B, Tu D, Pater J (2005). Analysis and interpretation of health-related quality-of-life data from clinical trials: Basic approach of the National Cancer Institute of Canada clinical trials group. European Journal of Cancer.

[20] Calvert M, Blazeby J, Altman DG (2013). Reporting of patient-reported outcomes in randomized trials: The CONSORT-PRO extension. JAMA.

[21] Efficace F, Fayers P, Pusic A, Cemal Y, Yanagawa J, Jacobs M (2015). Quality of patient-reported outcome reporting across cancer randomized controlled trials according to the CONSORT patient-reported outcome extension: A pooled analysis of 557 trials. Cancer.

[22] Bylicki O, Gan HK, Joly F, Maillet D, You B, Péron J (2014). Poor patient-reported outcomes reporting according to CONSORT guidelines in randomized clinical trials evaluating systemic cancer therapy. Annals of Oncology.

[23] Calvert M, Kyte D, von Hildebrand M, King M, Moher D (2015). Putting patients at the heart of health-care research. The Lancet.

[24] Calvert M, Kyte D, Duffy H, Gheorghe A, Mercieca-Bebber R, Ives J (2014). Patient reported outcome (PRO) assessment in clinical trials: A systematic review of guidance for trial protocol writers. Plos One.

[25] Mercieca-Bebber R, Friedlander M, Kok P-S, Calvert M, Kyte D, Stockler M (2016). The patient-reported outcome content of international ovarian cancer randomised controlled trial protocols. Quality of Life Research.

[26] Fairclough DL (2004). Patient reported outcomes as endpoints in medical research. Statistical Methods in Medical Research.

[27] Siegel RL, Miller KD, Jemal A (2016). Cancer statistics, 2016. CA: a Cancer Journal for Clinicians.

[28] Hanker LC, Loibl S, Burchardi N, Pfisterer J, Meier W, Pujade-Lauraine E (2012). The impact of second to sixth line therapy on survival of relapsed ovarian cancer after primary taxane/platinum-based therapy. Annals of Oncology.

[29] Mercieca-Bebber RL, Price M, Bell M, King MT, Webb P, PN Butow, et al. Ovarian cancer study dropouts had worse health-related quality of life and psychosocial symptoms at baseline and overtime. *Asia-Pacific Journal of Clinical Oncology*, doi:10.1111/ajco.12580 10.1111/ajco.12580 27573704

[30] Glasziou P, Altman DG, Bossuyt P, Boutron I, Clarke M, Julious S (2014). Reducing waste from incomplete or unusable reports of biomedical research. The Lancet.

[31] Bell ML, Fairclough DL (2014). Practical and statistical issues in missing data for longitudinal patient-reported outcomes. Statistical Methods in Medical Research.

[32] Schandelmaier, S., Conen, K., von Elm, E., You, J. J., Blumle, A., Tomonaga, Y., et al. (2015). Planning and reporting of quality-of-life outcomes in cancer trials. *Annals of Oncology, 26*(9), 1966–1973. Epub 2015 Jun 30. doi:10.1093/annonc/mdv283 10.1093/annonc/mdv283 PMC4551161 26133966

[33] Chalmers I, Glasziou P (2009). Avoidable waste in the production and reporting of research evidence. The Lancet.

[34] Rock EP, Kennedy DL, Furness MH, Pierce WF, Pazdur R, Burke LB (2007). Patient-reported outcomes supporting anticancer product approvals. Journal of Clinical Oncology.

[35] Gondek K, Sagnier P-P, Gilchrist K, Woolley JM (2007). Current status of patient-reported outcomes in industry-sponsored oncology clinical trials and product labels. Journal of Clinical Oncology.

[36] Ahmed, K., Kyte, D., Keeley, T., Efficace, F., Armes, J., Brown, J. M., et al. (2016). A systematic evaluation of patient-reported outcome (PRO) protocol content and reporting in UK cancer clinical trials: The EPiC study protocol. *BMJ Open, 6*. 10.1136/bmjopen-2016-012863 PMC5051436 27655263

