# Supplementary material for: A systematic evaluation of compliance and reporting of patient-reported outcome endpoints in ovarian cancer randomised controlled trials: implications for generalisability and clinical practice
Source: J Patient Rep Outcomes. 2017 Oct 4;1:5. doi: 10.1186/s41687-017-0008-3 (PMC5934909; doi:10.1186/s41687-017-0008-3)
Supplement: Supplementary file 1 — PRO protocol checklist CONSORT-PRO scoring sheets. (DOCX 27 kb) [file 41687_2017_8_MOESM1_ESM.docx]

**Appendix 1. PRO protocol checklist and CONSORT-PRO items scoring criteria**

**1a. PRO protocol checklist items**

|  | **PRO Protocol checklist item** |
| --- | --- |
| P1 | List **personnel responsible for PRO** components of trial |
| P2 | Describe **what is currently known about PROs** in this area and explain the gaps in literature |
| P3 | Provide a **rationale for the inclusion of PROs** as appropriate to the study population, intervention, context, objectives and setting |
| P4 | State the **PRO study objective** in relation to PRO domain/s, patient population and timeframe |
| P5 | State the **PRO hypothesis** & corresponding null hypothesis and to which outcome(s) the hypothesis relates |
|  | **Methods** |
| P6* | If PROs will be collected in a subset of the study population or in specific centres, include a description/rationale for the **sampling method** |
| P7 | State the **inclusion/exclusion criteria for PRO** endpoint(s) (e.g., language/reading requirements) |
| P8 | Specify if **PRO completion is pre-randomisation** eligibility requirement |
| P9 | Identify the **PRO endpoint as the primary, secondary** (and if so - whether a key/important secondary), or an exploratory endpoint |
| P10 | Describe the **PRO constructs** used to evaluate the intervention e.g. overall QOL, specific domain, specific symptom |
| P11 | Specify the **timepoint(s) for PRO analysis** (including the principle timepoint of interest) and provide the rationale for these |
| P12 | Include **PRO assessments** in the main protocol schedule of assessments, specifying which PRO measures (PROMs) will be used at each assessment |
| P13 | Specify if **baseline PRO assessment should be completed before randomisation** |
| P14 | Specify the targeted time and acceptable **time windows** for each PRO assessment |
| P15 | If PROs are to be completed in the clinic: **specify timing of PROM delivery** in relation to clinical assessments (e.g. before/whilst/after seeing clinician and/or clinical assessments) |
| P16 | **Justify the timing of PRO assessments**. Scheduled PRO assessments should link to research questions, hypotheses, length of recall, disease/treatment natural history, planned analysis and time of comparison must be comparable for both arms |
| P17^ | If PRO is the primary endpoint, state the required PRO **sample size,** otherwise discuss the power of the PRO analysis |
|  | **PROM & administration** |
| P18 | **Describe the PROMs** including, number of items/domains, instrument scaling/scoring, reliability, content and construct validity, responsiveness, sensitivity, acceptability, recall period. Provide references as appropriate |
| P19 | **Justify choice of PROM**(s) by linking specific domains/items to clinical justifications and hypotheses |
| P20 | Provide **evidence of measurement equivalence across modes** (i.e., when mixing modes of PRO data collection) and/or **of cross cultural validity** where different language versions of questionnaires are used |
| P21* | **Outline plans for evaluation of measurement properties, if appropriate**  (e.g. if not previously validated in the population of interest) |
| P22 | Specify the **estimated time to complete each assessment**, and discuss feasibility of assessment for the population" |
| P23 | Include a pre-specified **data collection plan** |
| P24 | Specify **how PROM will be completed** (e.g. pencil and paper, online, etc) |
| P25 | Specify **where PROM will be completed** (e.g. clinic, home, etc) |
| P26* | Where applicable, **justify use of proxie**s (define conditions under which proxy assessment is permissible) |
| P27 | Specify **who will administer the PROM** (e.g., a physician, nurse, etc) |
| P28 | If it is permissible for another person to help the study participant complete the PROM, describe what type **and level of assistance is acceptable** |
| P29* | If more than one PROM will be used, **specify whether the order of administration will be standardised or randomised** |
| P30 | Include a **plan for systematically training** and contacting local site personnel to ensure that they understand the content and importance of collecting PRO data. Ideally coordinated by a lead data manager who monitors PRO completion rates in real time and communicates with sites if completion rates are suboptimal |
| P31 | Specify procedures for **data collection and management methods to minimise missing data**. E.g. checking completed PROMs (including who will check forms and how will they deal with missing PROMs or missing items). |
| P32 | Include **guidance on discussing importance of PROs with patient** |
| P33 | Establish process for PRO assessment at (and beyond) **withdrawal for patients who withdraw early** from a study or who go 'off-study'/'off treatment' |
| P34 | Specify that a named person/position at each centre (and/or centrally) be nominated to take r**esponsibility for administration**, collection and checking of PROM - specify whether this is or is not the treating clinician |
|  | **Data management** |
| P35 | Specify how an e**lectronic PRO system/database will be maintained** and how investigator will meet regulatory requirements and ensure data integrity and security |
| P36 | Specify **plan to monitor PRO compliance,** including adherence to time windows |
| P37 | Include an **overview of PRO administration** (data collection), and d**ata handling/transmission and storage procedures** |
| P38 | Ensure **plans for administration of PROM(s)** are consistent with each PROM's user manual |
|  | **Analyses** |
| P39 | Include an a priori description of all **planned PRO analyses** pertaining to the study hypotheses |
| P40 | State the **assumptions of PRO analyses** |
| P41 | State the **anticipated response rate** and implications for the sample size |
| P42 | Include an a priori estimation of **PRO effect size** |
| P43 | S**pecify intention-to-treat** or per-protocol PRO analyses. |
| P44 | Include a prior**i identified summary statistics** (as appropriate) |
| P45 | Specify the **minimum PRO response rate** and acceptable degree of timing deviation (i.e acceptable time windows for each PRO assessment timepoint) before the PRO objective is compromised |
| P46 | Describe **methods for scoring endpoints.** Where possible, **reference scoring manuals** for summated scales from PROM (domain-specific &/or total) & **methods for handling missing items,** and methodological papers for composite endpoints (e.g. QTWiST) |
| P47 | State **statistical significance levels** and include plans for multiplicity/controlling type 1 error |
| P48 | **Pre-specify sequence of testing/exploratory analyses to control for multiplicit**y or pre-specify domains (e.g. in a regulatory trial/labelling claim) (Common in pharma trials. Involves pre-specifying domains that alpha would be spent on, or ordering the domains in priority & alpha would be spent down the list) |
| P49 | Specify the **criteria for clinical significance** (e.g. state minimal [clinical] important difference and/or responder definition (size and duration of benefit)) |
| P50 | State how **missing data will be described** |
| P51 | Describe method for **handling missing assessments** (e.g. approach to imputation and sensitivity analyses) |
|  | **Monitoring** |
| P52 | Describe the **role of the Data Monitoring Committee** and Quality Assurance for PROs |
| P53 | Include an a priori plan for consistent/standardised **management of PRO alerts** (symptoms/issues reported by patients that exceed a pre-defined level of severity) to be clearly communicated to all appropriate trial staff |
| P54 | Describe i**nformed consent procedure** for PRO assessment. |
| P55 | Specify whether **PRO forms will be used to influence therapy or patient management** |
| P56 | Include detailed **plans for regular feedback to participants** via letter/newsletter on PRO aspect of study |
|  | **General Approach to Protocol** |
| P57 | Identify PRO sections of the protocol in the **table of contents** |
| P58 | Provide **references** to support key PRO statements |

*This item was N/A for some or all protocols. ^We assessed all protocols for this item regardless of PRO endpoint status

**Appendix 1b. CONSORT-PRO review criteria & scoring**

Adapted from: Calvert M, Blazeby J, Altman DG, et al. Reporting of patient-reported outcomes in randomized trials: The CONSORT-PRO extension. JAMA 2013; 309: 814-822.

**P1b. Abstract – PRO noted as primary/secondary endpoint**

(1= yes, 0.5= PRO mentioned but unclear endpoint status; 0=no)

**2a. Rationale for including PROs**

(1= yes, 0=no)

**P2bi. PRO hypothesis present**

(0.5= yes, 0=no)

**P2bii. PRO domains specified in hypothesis**

(0.5= yes, 0=no)

**P6ai. Evidence of PRO instrument validity provided/cited**

(1= yes, 0=no)

**P6aii. Statement of the person completing the PRO measure (e.g. 'patients completed', or 'self-report')**

(0.5= yes, 0=no)

**P6aiii. Mode of administration specified (e.g. paper, e-PRO)**

(0.5= yes, 0=no)

**P12a. Statistical approach for dealing with missing data specified (e.g. imputation, omission of cases with missing data)**

(1= yes, 0=no)

**13ai. Report number of questionnaires submitted/available for analysis at baseline**

(0.5= yes, 0=no)

**13aii. Report number of questionnaires submitted/available for analysis principle timepoint for PRO analysis**

(0.5= yes, 0=no)

**15. Demographics table includes baseline PRO findings**

(1= yes, 0=no)

**16. Number of patients (denominator) included in each PRO analysis and whether this was intention to treat**

(1= yes, 0=no)

**17ai. PRO results reported for the hypothesised domains and time point specified in the hypothesis –OR- reported for each domain of the PROM if no PRO hypothesis provided**

(0.5= yes, 0=no)

**17aii. Results include confidence intervals, effect size or some other estimate of precision**

(0.5= yes, 0=no)

**18. Results of any subgroup/adjusted/exploratory analyses are reported**

(1=yes, 0=no)

**P20. PRO study limitations provided**

(1=yes, 0=no)

**P21. Implications of PRO results for generalizability, use in clinical practice**

(1=yes, 0=no)

**22. PROs interpreted in relation to clinical outcomes**

(1=yes, 0=no)

The CONSORT-PRO Extension was adapted for scoring as follows: CONSORT-PRO item 7a is relevant only to RCTs with primary PRO endpoint and was excluded from our scoring as only 1 RCT in our sample (n=36) had a co-primary endpoint. CONSORT-PRO items P2b, P6, 13a, 17a, P20/21 include multiple recommendations and were each divided into separate sub-items (each worth a score of 0.5) for the evaluation. RCTs were awarded a score of 0.5 or 1 (as indicated above) if the RCT addressed that item in the publication. The highest possible total CONSORT-PRO reporting score was 14.
