# Supplementary material for: A systematic evaluation of compliance and reporting of patient-reported outcome endpoints in ovarian cancer randomised controlled trials: implications for generalisability and clinical practice
Source: J Patient Rep Outcomes. 2017 Oct 4;1:5. doi: 10.1186/s41687-017-0008-3 (PMC5934909; doi:10.1186/s41687-017-0008-3)
Supplement: Supplementary file 2 — List of Included RCTs. (DOCX 21 kb) [file 41687_2017_8_MOESM2_ESM.docx]

**Appendix 2. Included RCTs**

1. Armstrong DK, Bundy B, Wenzel L et al. Intraperitoneal cisplatin and paclitaxel in ovarian cancer. New England Journal of Medicine 2006; 354: 34-43.

Wenzel LB, Huang HQ, Armstrong DK, Walker JL, Cella D, Gynecologic Oncology G. Health-related quality of life during and after intraperitoneal versus intravenous chemotherapy for optimally debulked ovarian cancer: a Gynecologic Oncology Group Study. J Clin Oncol. 2007;25(4):437-43.

1. Banerjee S, Rustin G, Paul J et al. A multicenter, randomized trial of flat dosing versus intrapatient dose escalation of single-agent carboplatin as first-line chemotherapy for advanced ovarian cancer: an SGCTG (SCOTROC 4) and ANZGOG study on behalf of GCIG. Ann Oncol. 2013; 24: 679-687. doi: 610.1093/annonc/mds1494. Epub 2012 Oct 1095.
2. Berek JS, Taylor PT, Gordon A et al. Randomized, placebo-controlled study of oregovomab for consolidation of clinical remission in patients with advanced ovarian cancer. Journal of Clinical Oncology 2004; 22: 3507-3516.
3. Bezjak et al. Quality of Life in Ovarian Cancer Patients: Comparison of Paclitaxel Plus Cisplatin, With Cyclophosphamide Plus Cisplatin in a Randomized Study. JCO 2004 22 (22) 4595-4603

   NCIC CTG Ov10 and EORTC 55931: Randomized Intergroup trial of cisplatinpaclitaxel versus cisplatin-cyclophosphamide in women with advanced ovarian cancer. (Lead author Piccart, published in JNCI 2000).

   Piccart M, Bertelsen K, Stuart G, et al: Long-term follow-up confirms a survival advantage of the paclitaxel-cisplatin regimen over the cyclophosphamide-cisplatin combination in advanced ovarian cancer. Ann Oncol 13:109, 2002(suppl 5).
4. Brundage et al. Health-related quality of life in recurrent platinum sensitive ovarian cancer - results from the CALYPSO trial. Annals of Oncology 23: 2020–2027, 2012

   Pujade-Lauraine E, Wagner U, Aavall-Lundqvist E et al. Pegylated liposomal Doxorubicin and Carboplatin compared with Paclitaxel and Carboplatin for patients with platinum-sensitive ovarian cancer in late relapse. Journal of Clinical Oncology 2010; 28: 3323-3329.
5. Colombo et al. Randomized, Open-Label, Phase III Study Comparing Patupilone (EPO906) With Pegylated Liposomal Doxorubicin in Platinum-Refractory or -Resistant Patients With Recurrent Epithelial Ovarian, Primary Fallopian Tube, or Primary Peritoneal Cancer. JCO November 1, 2012 vol. 30 no. 31 3841-3847
6. du Bois A, Floquet A, Kim J-W et al. Incorporation of pazopanib in maintenance therapy of ovarian cancer. Journal of Clinical Oncology 2014; 32: 3374-3382
7. du Bois A, Herrstedt J, Hardy-Bessard A-C et al. Phase III trial of carboplatin plus paclitaxel with or without gemcitabine in first-line treatment of epithelial ovarian cancer. Journal of Clinical Oncology 2010; 28: 4162-4169.
8. du Bois A, Weber B, Rochon J et al. Addition of epirubicin as a third drug to carboplatin-paclitaxel in first-line treatment of advanced ovarian cancer: a prospectively randomized gynecologic cancer intergroup trial by the Arbeitsgemeinschaft Gynaekologische Onkologie Ovarian Cancer Study Group and the Groupe d'Investigateurs Nationaux pour l'Etude des Cancers Ovariens. Journal of Clinical Oncology 2006; 24: 1127-1135.
9. Ferrandina 2008 Phase III Trial of Gemcitabine Compared With Pegylated Liposomal Doxorubicin in Progressive or Recurrent Ovarian Cancer. J Clin Oncol. 2008 Feb 20;26(6):890-6. doi: 10.1200/JCO.2007.13.6606.
10. Fotopoulo C et al. Weekly carboplatin in acq platinum resistant ovarian cancer w/wo oral phenoxodiol, a sensitizer of platinum cytotoxicity: the phase3 OVATURE multicentre randomised trial Ann Oncol. 2014 Jan;25(1):160-5. doi: 10.1093/annonc/mdt515. Epub 2013 Dec 5.
11. Greimel ER, Bjelic-Radisic V, Pfisterer J et al. Randomized study of the Arbeitsgemeinschaft Gynaekologische Onkologie Ovarian Cancer Study Group comparing quality of life in patients with ovarian cancer treated with cisplatin/paclitaxel versus carboplatin/paclitaxel. Journal of Clinical Oncology 2006; 24: 579-586.

    du Bois A, Luck H-J, Meier W et al. A randomized clinical trial of cisplatin/paclitaxel versus carboplatin/paclitaxel as first-line treatment of ovarian cancer. Journal of the National Cancer Institute 2003; 95: 1320-1329.
12. Harano K, Terauchi F, Katsumata N, Takahashi F, Yasuda M, Takakura S, et al. Quality-of-life outcomes from a randomized phase III trial of dose-dense weekly paclitaxel and carboplatin compared with conventional paclitaxel and carboplatin as a first-line treatment for stage II-IV ovarian cancer: Japanese Gynecologic Oncology Group Trial (JGOG3016). Ann Oncol. 2014;25(1):251-7.

Katsumata N, Yasuda M, Takahashi F et al. Dose-dense paclitaxel once a week in combination with carboplatin every 3 weeks for advanced ovarian cancer: a phase 3, open-label, randomised controlled trial. Lancet 2009; 374: 1331–1338.

1. Hirte et al. A phase III randomized trial of BAY 12-9566 (tanomastat) as maintenance therapy in patients with advanced ovarian cancer responsive to primary surgery and paclitaxel/platinum containing chemotherapy: A National Cancer Institute of Canada Clinical Trials Group Study. Gynae Oncology 2006
2. Krasner et al Patient-reported outcomes in relapsed ovarian cancer: Results from a randomizedPhase III study of trabectedin with pegylated liposomal doxorubicin (PLD) versus PLD Alone
3. Ledermann et al. Cediranib in patients with relapsed platinum-sensitive ovarian cancer (ICON6): a randomised, double-blind, placebo-controlled phase 3 trial. The Lancet. Volume 387, Issue 10023, 12–18 March 2016, Pages 1066–1074
4. Ledermann J, Harter P, Gourley C et al. Olaparib maintenance therapy in platinum-sensitive relapsed ovarian cancer. New England Journal of Medicine 2012; 366: 1382-1392.
5. Lindemann K, Christensen RD, Vergote I et al. First-line treatment of advanced ovarian cancer with paclitaxel/carboplatin with or without epirubicin (TEC versus TC)--a gynecologic cancer intergroup study of the NSGO, EORTC GCG and NCIC CTG. Annals of Oncology 2012; 23: 2613-2619.
6. Monk BJ, Poveda A, Vergote I, Raspagliesi F, Fujiwara K, Bae D-S, et al. Anti-angiopoietin therapy with trebananib for recurrent ovarian cancer (TRINOVA-1): a randomised, multicentre, double-blind, placebo-controlled phase 3 trial. Lancet Oncol. 2014;15(8):799-808
7. Monk et al. Patient reported outcomes of a randomized, placebo-controlled trial of bevacizumab in the front-line treatment of ovarian cancer: A Gynecologic Oncology Group Study. Gynecologic Oncology128 (2013) 537-578

   Burger et al. Incorporation of Bevacizumab in the Primary Treatment of Ovarian Cancer. The New England Journal of Medicine 2011: 365:2473-83.

Chase DM, Huang H, Foss CD, Wenzel LB, Monk BJ, Burger RA. Neurotoxicity in ovarian cancer patients on Gynecologic Oncology Group (GOG) protocol 218: characteristics associated with toxicity and the effect of substitution with docetaxel: an NRG Oncology/Gynecologic Oncology Group study. Gynecol Oncol. 2015;136(2):323-7.

1. Mutch DG et al. Randomized Phase III Trial of Gemcitabine Compared With Pegylated Liposomal Doxorubicin in Patients With Platinum-Resistant Ovarian Cancer. J Clin Oncol. 2007 Jul 1;25(19):2811-8.
2. Pfisterer J, Weber B, Reuss A et al. Randomized phase III trial of topotecan following carboplatin and paclitaxel in first-line treatment of advanced ovarian cancer: a gynecologic cancer intergroup trial of the AGO-OVAR and GINECO. Journal of the National Cancer Institute 2006; 98: 1036-1045.
3. Pfisterer, et al. Gemcitabine Plus Carboplatin Compared With Carboplatin in Patients With Platinum-Sensitive Recurrent Ovarian Cancer: An Intergroup Trial of the AGO-OVAR, the NCIC CTG, and the EORTC GCG. JCO 2006

   Pfisterer J, Vergote I, Du Bois A et al. Combination therapy with gemcitabine and carboplatin in recurrent ovarian cancer. International Journal of Gynecological Cancer 2005; 15 Suppl 1: 36-41.
4. Pignata S, Scambia G, Ferrandina G et al. Carboplatin plus paclitaxel versus carboplatin plus pegylated liposomal doxorubicin as first-line treatment for patients with ovarian cancer: the MITO-2 randomized phase III trial. Journal of Clinical Oncology 2011; 29: 3628-3635.
5. Pignata S, Scambia G, Katsaros D et al. Carboplatin plus paclitaxel once a week versus every 3 weeks in patients with advanced ovarian cancer (MITO-7): a randomised, multicentre, open-label, phase 3 trial. Lancet Oncology 2014; 15: 396-40
6. Reed NS, Poole CJ, Coleman R et al. A randomised comparison of treosulfan and carboplatin in patients with ovarian cancer: a study by the Scottish Gynaecological Cancer Trials Group (SGCTG). Eur J Cancer. 2006; 42: 179-185. Epub 2005 Dec 2006.
7. Rustin, Van der Burg, griffin, Lamont, Jayson et al. Early vs Delayed treatment of relapsed ovarian cancer MRC OV05/EORTC 55955. Lancet 2010;376:1155-63.
8. Sehouli 2008 Nonplatinum Topotecan Combinations Versus Topotecan Alone for Recurrent Ovarian Cancer: Results of a Phase III Study of the North-Eastern German Society ofGynecological Oncology Ovarian Cancer Study Group, J Clin Oncol 26:3176-3182.
9. Stark D, Nankivell M, Pujade-Lauraine E, Kristensen G, Elit L, Stockler M, Hilpert F, Cervantes A, Brown J, Lanceley A, Velikova G, Sabate E, Pfisterer J, Carey MS, Beale P, Qian W, Swart AM, Oza A, Perren T. Standard chemotherapy with or without bevacizumab in advanced ovarian cancer: quality-of-life outcomes from the International Collaboration on Ovarian Neoplasms (ICON7) phase 3 randomised trial. Lancet Oncol. 2013 Mar;14(3):236-43. doi: 10.1016/S1470-2045(12)70567-3. Epub 2013 Jan 18.
   Perren et al. A Phase 3 Trial of Bevacizumab in Ovarian Cancer . NEJM 365;26 2011
10. Stockler MR, Hilpert F, Friedlander M, King MT, Wenzel L, Lee CK, Joly F, de Gregorio N, Arranz JA, Mirza MR, Sorio R, Freudensprung U, Sneller V, Hales G, Pujade-Lauraine E.Patient-reported outcome results from the open-label phase III AURELIA trial evaluating bevacizumab-containing therapy for platinum-resistant ovarian cancer.J Clin Oncol. 2014 May 1;32(13):1309-16.

    Pujade-Lauraine et al. Bevacizumab Combined With Chemotherapy for Platinum-Resistant Recurrent Ovarian Cancer: The AURELIA Open-Label Randomized Phase III Trial. DOI: 10.1200/JCO.2013.51.4489 Journal of Clinical Oncology 32, no. 13 (May 2014) 1302-1308.
11. ten Bokkel Huinink, et al. Long Term survival in a Phase 3 randomised study of topotecan versus paclitaxel in advanced epithelial ovarian cancer (international topotecan study group)Ann Oncol. 2004 Jan;15(1):100-3.
12. The ICON and AGO Collaborators. Paclitaxel plus platinum-based chemotherapy versus conventional platinum-based chemotherapy in women withrelapsed ovarian cancer: the ICON4/AGO-OVAR-2.2 trial. Lancet 2003; 361: 2099–106
13. Vasey PA, Jayson GC, Gordon A et al. Phase III randomized trial of docetaxel-carboplatin versus paclitaxel-carboplatin as first-line chemotherapy for ovarian carcinoma. Journal of the National Cancer Institute 2004; 96: 1682-1691.
14. Vergote et al. Neoadjuvant chemotherapy or primary surgery in Stage IIIC or IV ovarian cancer (EORTC GCG, NCIC CTG, GCIC).N Engl J Med. 2010 Sep 2;363(10):943-53. doi: 10.1056/NEJMoa0908806.
15. Vergote IB, Jimeno A, Joly F et al. Randomized phase III study of erlotinib versus observation in patients with no evidence of disease progression after first-line platin-based chemotherapy for ovarian carcinoma: a European Organisation for Research and Treatment of Cancer-Gynaecological Cancer Group, and Gynecologic Cancer Intergroup study. Journal of Clinical Oncology 2014; 32: 320-326.
16. Wenzel L, Huang HQ, Monk BJ, Rose PG, Cella D. Quality-of-life comparisons in a randomized trial of interval secondary cytoreduction in advanced ovarian carcinoma: a Gynecologic Oncology Group study. J Clin Oncol. 2005;23(24):5605-12.
