# Supplementary material for: A systematic evaluation of compliance and reporting of patient-reported outcome endpoints in ovarian cancer randomised controlled trials: implications for generalisability and clinical practice
Source: J Patient Rep Outcomes. 2017 Oct 4;1:5. doi: 10.1186/s41687-017-0008-3 (PMC5934909; doi:10.1186/s41687-017-0008-3)
Supplement: Supplementary file 3 — Supplementary plots. (DOCX 135 kb) [file 41687_2017_8_MOESM3_ESM.docx]

**Appendix 3. Supplementary plots**

**3a. Scatterplot of total CONSORT-PRO score by year of 36 ovarian cancer RCTs**

**3b. Dot plot of total CONSORT-PRO score comparing RCTs that reported a significant difference in PRO endpoint to RCTs which did not report a significant difference (n=36)**


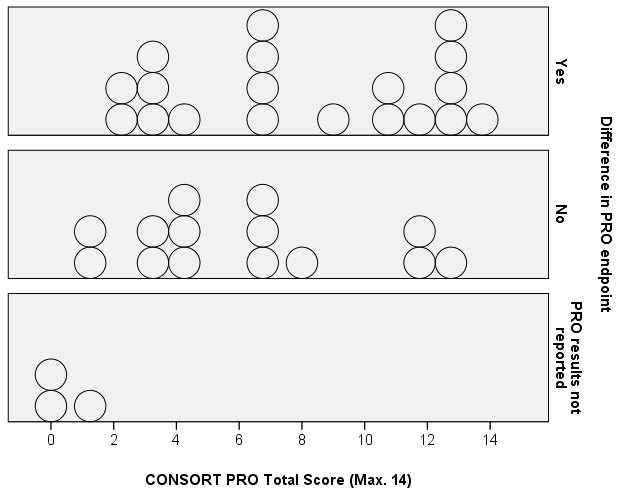


**3c. Dot plot comparing PRO protocol checklist total score by whether any PRO compliance information was reported (n=26*)**


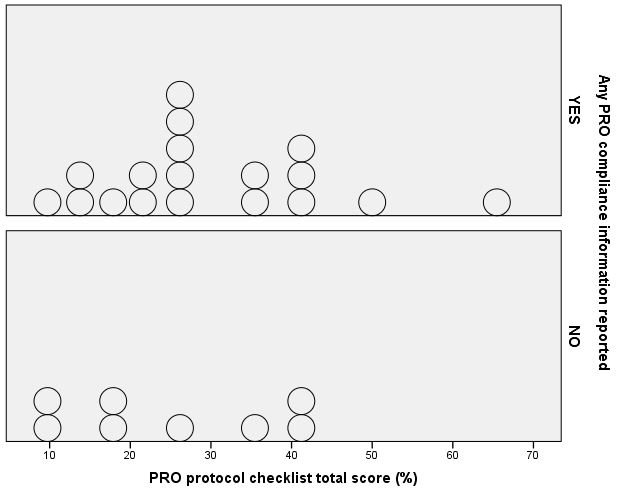


*Note: The protocols of 10/36 RCTs included in this study were not available for analysis and are excluded from this plot.

**3d. CONSORT-PRO scores for RCTs that reported some PRO compliance data VS RCTs that reported none**

**
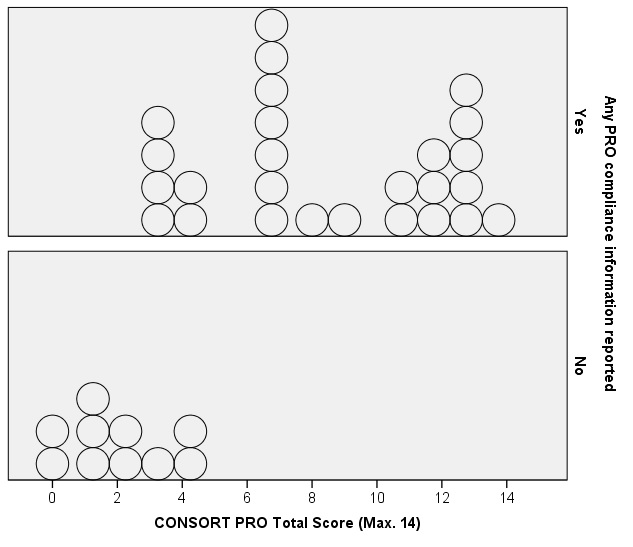
**
